# Intracoronary versus intravenous abciximab in ST-segment elevation myocardial infarction: rationale and design of the CICERO trial in patients undergoing primary percutaneous coronary intervention with thrombus aspiration

**DOI:** 10.1186/1745-6215-10-90

**Published:** 2009-09-28

**Authors:** Youlan L Gu, Marieke L Fokkema, Marthe A Kampinga, Bart JGL de Smet, Eng S Tan, Ad FM van den Heuvel, Felix Zijlstra

**Affiliations:** 1 Department of Cardiology, Thorax Center, University Medical Center Groningen, University of Groningen, Hanzeplein 1 PO Box 30001, 9700 RB Groningen, the Netherlands

## Abstract

**Background:**

Administration of abciximab during primary percutaneous coronary intervention is an effective adjunctive therapy in the treatment of patients with ST-segment elevation myocardial infarction. Recent small-scaled studies have suggested that intracoronary administration of abciximab during primary percutaneous coronary intervention is superior to conventional intravenous administration. This study has been designed to investigate whether intracoronary bolus administration of abciximab is more effective than intravenous bolus administration in improving myocardial perfusion in patients with ST-segment elevation myocardial infarction undergoing primary percutaneous coronary intervention with thrombus aspiration.

**Methods/Design:**

The Comparison of IntraCoronary versus intravenous abciximab administration during Emergency Reperfusion Of ST-segment elevation myocardial infarction (CICERO) trial is a single-center, prospective, randomized open-label trial with blinded evaluation of endpoints. A total of 530 patients with STEMI undergoing primary percutaneous coronary intervention are randomly assigned to either an intracoronary or intravenous bolus of weight-adjusted abciximab. The primary end point is the incidence of >70% ST-segment elevation resolution. Secondary end points consist of post-procedural residual ST-segment deviation, myocardial blush grade, distal embolization, enzymatic infarct size, in-hospital bleeding, and clinical outcome at 30 days and 1 year.

**Discussion:**

The CICERO trial is the first clinical trial to date to verify the effect of intracoronary versus intravenous administration of abciximab on myocardial perfusion in patients with ST-segment elevation myocardial infarction undergoing primary percutaneous coronary intervention with thrombus aspiration.

**Trial registration:**

ClinicalTrials.gov NCT00927615

## Background

ST-segment elevation myocardial infarction (STEMI) is generally caused by rupture or erosion of atherosclerotic plaque and subsequent platelet aggregation and thrombosis, resulting in acute occlusion of a coronary artery [1,2]. The preferred treatment strategy consists of prompt reperfusion therapy by means of primary percutaneous coronary intervention (PCI) [3-5]. However, despite optimal reperfusion of the infarct-related coronary artery, impaired myocardial perfusion is still present in a significant proportion of patients following successful PCI, which is associated with larger infarct size and increased long-term cardiac mortality [6,7].

One of the major causes of impaired myocardial reperfusion is embolization of atherothrombotic material including platelet aggregates into the distal microcirculation [8]. In recent years, the implementation of adjunctive mechanical and pharmacological therapies during primary PCI, including manual thrombus aspiration and glycoprotein (GP) IIb/IIIa inhibitors, has significantly reduced the occurrence of distal embolization and improved clinical outcome in STEMI patients [9-15]. Several trials and meta-analyses have demonstrated that manual thrombus aspiration improved myocardial reperfusion in patients presenting with STEMI and was associated with improved survival compared to conventional PCI at clinical follow-up up to 1 year [11,12,16-20]. However, a major limitation of thrombus aspiration is its inability to prevent microvascular obstruction that has occurred prior to PCI or that has been induced by primary PCI including thrombus aspiration itself. Adjunctive pharmacological therapies are therefore needed to target these sources of microvascular obstruction.

Anti-platelet therapy is an important cornerstone of modern STEMI management. During PCI, the use of GP IIb/IIIa inhibitors improves microvascular reperfusion [13,14]. In large randomized trials, intravenous (IV) administration of the GPIIb/IIIa inhibitor abciximab during PCI was associated with a significant reduction in short- and long-term mortality and reinfarction rates in patients with STEMI [9,10,15]. An alternative approach with the use of bivalirudin instead of the combination of unfractionated heparin and a GPIIb/IIIa inhibitor has been advocated and investigated [21]. Although this may result in a lower rate of bleeding complications, a major drawback seems to be the higher incidence of stent thrombosis.

Abciximab is the Fab fragment of the chimeric monoclonal antibody 7E3, which acts as a potent platelet aggregation inhibitor mainly by binding to the GP IIb/IIIa receptor on the surface of activated human platelets. Hereby, abciximab inhibits the final common pathway for platelet aggregation by preventing the binding of fibrinogen and von Willebrand factor to activated platelets [22]. A receptor occupancy study reported that the absolute number of free GP IIb/IIIa receptors was decreased in patients with successful restoration of myocardial perfusion who were treated with GP IIb/IIIa inhibitors [23]. Experimental studies have reported additional dose-dependent anti-platelet and anti-thrombotic effects of abciximab, which is not only able to prevent thrombus formation, but also to facilitate the dispersal of newly formed platelet aggregates by displacement of platelet bound fibrinogen with higher local drug concentration, and to inhibit platelet-induced thrombin generation [22,24,25]. In contrast to other GP IIb/IIIa inhibitors, abciximab has also distinct non-GP IIb/IIIa-related properties that may reduce inflammatory pathways and reperfusion injury [26]. These dose-dependent anti-platelet, anti-thrombotic and anti-inflammatory features of abciximab suggest that a higher local platelet inhibitor concentration may translate into further clinical improvements. Higher local concentrations can be obtained by the direct administration of abciximab into the infarct-related artery.

Intracoronary (IC) administration of abciximab has been investigated in several case reports and clinical studies [27-35] (Table 1). A retrospective study suggested a significant clinical benefit of IC administration, showing a 50% reduction of major adverse cardiac events (MACE) in patients with acute coronary syndromes treated with IC abciximab compared to IV abciximab [34]. A small prospective randomized trial showed a greater degree of myocardial salvage, better recovery of left ventricular function, and improved myocardial perfusion in patients with STEMI treated with IC abciximab [27]. Thiele et al [33] reported a reduced infarct size and extent of microvascular obstruction, and improved perfusion in patients treated with an IC bolus of abciximab. In addition, there was a trend towards a clinically relevant reduction in the incidence of MACE in patients treated with IC abciximab (5.2% vs. 15.6%, p = 0.06).

**Table 1:** Studies comparing IC and IV administration of abciximab in patients with STEMI

**Author**	Year*	**Design**	**Patients**	**No**	**In favor of**	**Main results**
Wohrle et al [34]	2003	retrospective	STEMI/NSTEACS	403	IC	reduced incidence of MACE at 30 days
Bellandi et al [27]	2004	prospective, randomized	STEMI	45	IC	higher salvage index and LV functional recovery (serial gated SPECT)
Romagnoli et al [32]	2005	prospective, matched	STEMI/NSTEACS	74	IC	increased coronary flow (cTFC)
Galache et al [29]	2006	prospective, randomized	STEMI/NSTEACS	137	neutral	no difference in the incidence of MACE at 1 year
Thiele et al [33]	2008	prospective, randomized	STEMI	154	IC	reduced infarct size and extent of MO (MRI at 2 days)
Dominguez-Rodriguez et al [28]	2009	prospective, randomized	STEMI	50	IC	larger reduction in soluble CD40 ligand

* Year of publication

cTFC: corrected TIMI frame count; IC: intracoronary; IV: intravenous; MACE: major adverse cardiac event; MO: microvascular obstruction; MRI: magnetic resonance imaging; No: number of patients; NSTEACS: non-ST-elevation acute coronary syndrome; SPECT: single photon emission computed tomography; STEMI: ST-segment elevation myocardial infarction

Given the limited number of patients included in these trials, a larger randomized clinical trial is required to verify the effect of IC abciximab administration in STEMI patients undergoing primary PCI. Furthermore, there is at the present time no information with regard to the combined strategy of thrombus aspiration and IC use of abciximab. Therefore, we intend to determine the effect of IC bolus administration of abciximab on post-procedural myocardial perfusion compared to IV bolus administration in STEMI patients undergoing primary PCI with thrombus aspiration.

## Methods/Design

**Figure 1:**
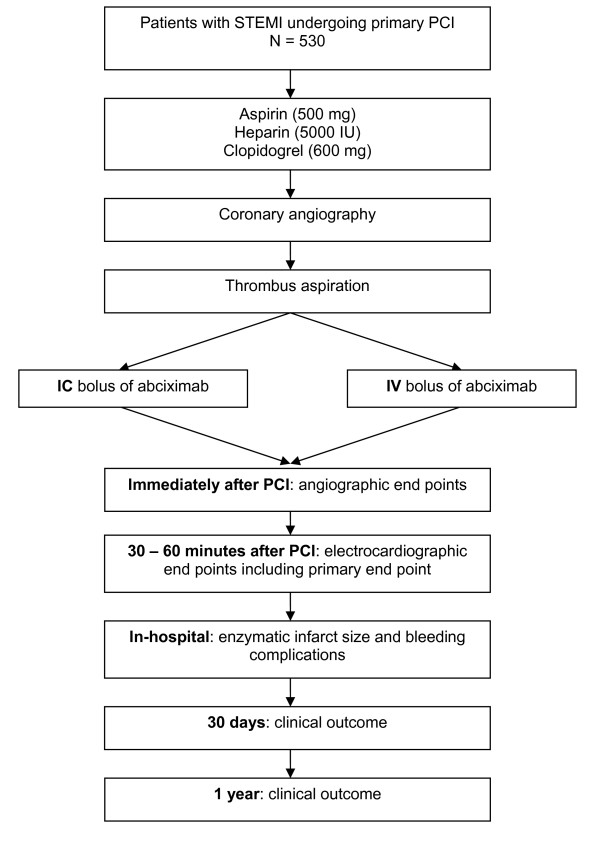
**The CICERO trial flow chart**. IC: intracoronary; IV: intravenous; PCI: percutaneous coronary intervention; STEMI: ST-segment elevation myocardial infarction.

The CICERO trial is a single-center, prospective, randomized trial with blinded evaluation of endpoints (Figure 1). A total of 530 patients with STEMI undergoing primary PCI are randomly assigned to either an IC or IV bolus of weight-adjusted abciximab (0.25 mg/kg body weight, ReoPro 2 mg/ml, Centocor B.V., Leiden, the Netherlands). Randomization is performed by means of sealed envelopes at the catheterization laboratory when a decision to perform PCI is taken. The study takes place at a high-volume university hospital center providing 24-hours emergency cardiac care with 7 referral hospitals in a region with 750,000 inhabitants. The study was approved by the institutional committee on human research of the University Medical Center of Groningen and is in compliance with the declaration of Helsinki. The protocol of this trial has been registered at ClinicalTrials.gov (NCT00927615).

### Study population

All consecutive STEMI patients who are candidates for primary PCI are considered eligible for participation. The inclusion criterion is a diagnosis of STEMI as defined by chest pain suggestive for myocardial ischemia for at least 30 minutes before hospital admission, time from onset of symptoms of less than 12 hours, and an ECG with new ST-segment elevation in 2 or more contiguous leads of ≥0.2 mV in leads V2-V3 and/or ≥0.1 mV in other leads or a new-onset left bundle branch block. Exclusion criteria are rescue PCI after thrombolytic therapy, need for emergency coronary artery bypass grafting, presence of cardiogenic shock, known existence of a life-threatening disease with a life expectancy of less than 6 months, inability to provide informed consent, age below 18 years, and contra-indications for the use of abciximab, which include active internal bleeding, history of stroke within 2 years, recent major surgery or intracranial or intraspinal trauma or surgery within 2 months, intracranial neoplasm, arteriovenous malformation or aneurysm, bleeding diathesis, severe uncontrolled hypertension, thrombocytopenia, vasculitis, hypertensive or diabetic retinopathy, severe liver or kidney failure, and hypersensitivity to murine proteins.

### Treatment

During PCI, the initial treatment step consists of manual thrombus aspiration whenever possible. Thrombus aspiration is performed with the Export aspiration catheter (Medtronic Inc., Santa Rosa, USA) as previously described [11]. Continuous manual suction is performed using a proximal-to-distal approach, which is defined as active aspiration during initial passage of the lesion. In patients assigned to IC administration, a bolus of abciximab is administered through the guiding catheter proximal to the lesion in the infarct-related artery over a period of 1 minute after restoration of anterograde flow. As final step a stent is implanted. Additional pre- or postdilatation with a balloon may be required in certain patients.

Patients are pre-treated with aspirin (500 mg), heparin (5000 IU IV), and high-dose clopidogrel (600 mg orally) after electrocardiographic confirmation of STEMI, usually in the ambulance. IC administration of nitroglycerine (400 μg) is administered during the procedure at the operator's discretion. During PCI, additional low-dose weight-adjusted heparin is administered as guided by the activated clotting time (target: 200 - 250 seconds). Sheaths are removed immediately at the end of the PCI procedure using the Angio-Seal device (St. Jude Medical, Inc, St. Paul, MN, USA) or by manual compression after arrival at the coronary care unit. In the setting of STEMI, the femoral approach is preferred. The radial approach is reserved for patients without femoral access. In case of radial access, sheaths are removed immediately following the PCI procedure. In patients with atrial fibrillation, a large dyskinetic area of the left ventricle, and in immobile patients, low-molecular-weight heparin is given for 1 to 3 days after sheath removal. Standard therapy after PCI includes aspirin (80 mg), clopidogrel (75 mg), beta-blockers, lipid lowering agents, and angiotensin converting enzyme inhibitors or angiotensin II receptor blockers, according to current international guidelines [36].

### Electrocardiography

A standard 12-lead electrocardiogram (ECG) is acquired at the time of presentation and at 30 to 60 minutes after the end of procedure. Times of onset of symptoms, admission, first intracoronary intervention, end of PCI and ECG recordings are registered. The magnitude of ST-segment deviation is measured 60 ms from J-point. The first post-intervention ECG at 30 to 60 minutes is classified by comparison of the ST-segments with those of the ECG at presentation. ST-segment elevation resolution is categorized as complete (>70%), partial (30-70%), or absent (<30%) [6]. On the post-interventional ECG, residual ST-segment deviation is calculated as the sum of residual ST-segment elevation and depression in all leads [37]. New-onset of Q waves on the post-interventional ECG is defined as an initial negative deflection of the QRS complex of >0.1 mV and >40 ms in an ECG lead related to the myocardial area of infarction together with all pathological Q waves. All ECG recordings are analyzed by a physician blinded to treatment allocation and clinical data.

### Coronary angiography

The following baseline, peri- and post-procedural angiographic features are recorded: the presence of thrombus and collaterals, Thrombolysis In Myocardial Infarction (TIMI) flow grades, myocardial blush grade (MBG), and the presence of angiographically visible distal embolization. TIMI flow grades are estimated as previously described [38]. Thrombus is assessed according to the criteria summarized by Mabin et al [39]. These criteria include the presence of an intraluminal central filling defect or lucency surrounded by contrast material that is seen in multiple projections, the absence of calcium within the defect; and persistence of contrast material within the lumen. Collaterals are assessed according to Rentrop's classification [40]: 0 = none, 1 = filling of side branches only, 2 = partial filling of the epicardial segment, 3 = complete filling of the epicardial segment. Evaluation of MBG is performed as described by van 't Hof et al [7]: 0 = no myocardial blush, 1 = minimal myocardial blush or contrast density, 2 = moderate myocardial blush or contrast density, but less than that obtained during angiography of a contra- or ipsilateral non-infarct-related coronary artery, and 3 = normal myocardial blush or contrast density, comparable with that obtained during angiography of a contra- or ipsilateral non-infarct-related coronary artery. Persisting myocardial blush ("staining") suggests leakage of contrast medium into the extravascular space and is graded 0. In addition, MBG is quantified with the Quantitative Blush Evaluator (QuBE) as described by Vogelzang et al [41], which provides a computer-assisted and more operator-independent score by calculating the increase and decrease of myocardial contrast density in the myocardial area of interest. Distal embolization is considered to have occurred if new circumscribed filling defects and/or abrupt cutoff of the vessel distal to the target lesion appears [42,43]. The coronary angiograms are analyzed by a physician who is blinded to treatment allocation and clinical data.

### Infarct size

Infarct size is estimated by serial measurements of cardiac markers including creatinine kinase (CK), myocardial band fraction of CK (CK-MB), lactate dehydrogenase (LDH), and troponin T. Blood is sampled at baseline and at 3, 6, 9, 12, 18, 24, and 48 hours after PCI. Peak release, time to peak release as well as area under the curve is determined. Marker levels are determined on a Hitachi 717 automatic analyzer according to the International Federation of Clinical Chemistry (IFCC) recommendation.

### End points assessment

The primary end point is the incidence of ST-segment resolution >70% as assessed on the ECG acquired 30 to 60 minutes after PCI compared to the ECG at presentation.

Secondary end points include:

- Angiographic end points: post-procedural TIMI flow, MBG (by visual estimation and with the QuBE program) and angiographically visible distal embolization

- Electrocardiographic end points: residual ST-segment deviation 30 to 60 minutes after the procedure

- Enzymatic infarct size

- Mortality and Major Adverse Cardiac Events (MACE, a combined end point of target vessel revascularization, reinfarction, and cardiovascular mortality) at 30 days and 1 year.

A safety endpoint consists of in-hospital bleeding complications.

Furthermore, the primary and secondary endpoints are to be analyzed in pre-specified subgroups, which are defined as:

1. Age (<65 versus >65 years)

2. Gender

3. Presence of diabetes

4. Number of diseased vessels (multi-vessel versus single vessel)

5. Infarct-related artery (left anterior descending artery (LAD) versus non-LAD)

6. Ischemic time (<3 versus >3 hours)

7. Angiographic presence of thrombus

8. Pre-procedural TIMI flow

9. Post-procedural TIMI flow

10. Post-procedural myocardial blush grade

### Clinical follow-up

Death, reinfarction, and ischemia driven target-vessel revascularization are to be registered at 30 days and 1 year. Follow-up information will be obtained from the central personal records database, hospital records as well as by telephone interviews with the patients and/or their general practitioners.

### Statistical considerations

#### Sample size estimation

In previously published data, the incidence of our primary end point resolution of ST-segment elevation >70% has been reported to be 56.6% in patients with STEMI treated with thrombus aspiration [11]. We hypothesize that IC administration of abciximab during PCI increases the incidence of ST-segment resolution >70% by 25%. To detect a 25% difference between the two treatment groups, 530 patients are required to reach a 5% significance level (two-sided) with 90% power.

#### Statistical analysis

All statistical analyses will be performed according to the intention-to-treat principle for the overall population as well as for the pre-specified subgroups. Statistical significance is considered as a two-tailed p value less than 0.05. The Statistical Package for the Social Sciences (SPSS Inc., Chicago, IL, USA) version 16.0.2 will be used for all statistical analyses. Differences between group means will be assessed with the two-tailed Student's t-test. Chi-square analysis or Fisher's exact test will be used to test differences between proportions. Survival will be calculated by the Kaplan-Meier product-limit method. Chi-square analysis will be used to assess the relation between an individual variable and end points. The Mantel-Cox (or log-rank) test will be used to evaluate differences in survival between the two treatment groups. The Cox proportional-hazards regression model will be used to calculate relative risks and to adjust for differences in baseline characteristics.

## Discussion

The CICERO trial is a single-center, prospective, randomized trial to determine whether IC administration of abciximab during primary PCI is more effective than IV administration in improving myocardial perfusion in STEMI patients undergoing primary PCI with thrombus aspiration. This is the first large clinical trial to date to determine the effect of IC versus IV administration of abciximab in STEMI patients undergoing primary PCI with thrombus aspiration.

## Competing interests

The authors declare that they have no competing interests.

## Authors' contributions

YLG designed the study in collaboration with MLF and FZ and drafted the manuscript. MAK, BJGLS, EST, AFMH, and FZ revised the manuscript critically for important intellectual content. All authors read and approved the final manuscript.
